# OnabotulinumtoxinA treatment for chronic migraine: experience in 52 patients treated with the PREEMPT paradigm

**DOI:** 10.1186/s40064-015-0957-z

**Published:** 2015-04-13

**Authors:** María Isabel Pedraza, Carolina de la Cruz, Marina Ruiz, Luis López-Mesonero, Elena Martínez, Mercedes de Lera, Ángel Luis Guerrero

**Affiliations:** Neurology Department, Hospital Clínico Universitario, Valladolid, Spain

**Keywords:** Migraine, Chronic migraine, OnabotulinumtoxinA, Topiramate

## Abstract

OnabotulinumtoxinA (OnabotA) was approved for treatment of chronic migraine (CM) after publication of PREEMPT trials. Thus, we set out to evaluate the efficacy of OnabotA in a series of patients with CM treated according to the PREEMPT protocol.

In May 2012 we began to offer OnabotA to patients with CM who did not respond to topiramate and at least one other preventive therapy (beta blocker and/or calcium channel antagonist). We prospectively recorded demographic data and the characteristics of migraine, and we assessed the modifications in monthly headache and migraine days, as well as the number of days of symptomatic medication and triptan intake.

By September 2014 we had treated 52 patients (8 male, 44 female), whose age at treatment onset was 42.8 ± 12.7 years (range: 16–71) and age at migraine onset was 16.8 ± 7.8 years (3–32). In 43 of these patients (82.7%) symptomatic overuse of medication was observed at the onset of treatment. A total of 168 procedures were performed and after the first session, we observed a significant reduction in all the variables considered. Twelve (23.1%) patients failed to perceive a positive effect after the first procedure and it was not repeated in 4 of them. By contrast, there was a significant decreasing in all the variables evaluated compared to the baseline in the 39 patients that received a second series of injections.

The use of OnabotA according to the PREEMPT paradigm is an effective treatment in patients with chronic migraine in a real-life setting.

## Background

Chronic migraine (CM) is a common and disabling condition affecting up to 2.4% of the population (Manack et al. 2011; Natoli et al. 2010). CM was recognized as an independent entity in the third edition of the International Classification of Headache Disorders (ICHD-III), where it was defined as persistent headache on ≥ 15 days per month for ≥ 3 months; the criteria for migraine were met or the headache responded to migraine-specific treatment on ≥ 8 days (Headache Classification Committee of the International Headache Society (IHS) 2013).

Risk factors for the chronification of migraine have been identified, such as female gender, older age, Caucasian ethnicity, low socio-economic status, co-morbidity with other chronic diseases like obesity or psychiatric disorders, high frequency episodic migraine and overuse of symptomatic medication (Natoli et al. 2010; Rojo et al. 2015).

After publication of the PREEMPT clinical study (Aurora et al. 2010; Diener et al. 2010; Dodick et al. 2010), in January 2012 OnabotulinumtoxinA (OnabotA) was licensed in Spain for prophylactic treatment of CM “for patients who have not adequately responded or are intolerant to prophylactic drugs for migraine”.

Our objective was to analyze the efficacy of OnabotA in treating CM in our population following the infiltration paradigm described in the PREEMPT studies (Blumenfeld et al. 2010) in a real clinical setting.

## Methods

From May 2012, OnabotA was offered to adult patients with CM (revised ICHD-II criteria) (Olessen et al. 2006) who attended a headache outpatient clinic at a tertiary hospital. We included patients who experienced a significant disruption in their quality of life, and who had not responded positively to previous treatment with topiramate (or another neuromodulator if topiramate was not tolerated) and at least one other preventative therapy (beta blocker and/or calcium channel antagonist), ensuring that all these drugs had been administered at adequate doses and for sufficient time to have been effective (Levin 2008; Schulman et al. 2008). In cases with tenderness to palpation of the pericranial nerves (occipital or supraorbital), at least one anaesthetic blockade was performed with no effect.

We collected the demographic data and the migraine characteristics from all the patients. We did not exclude patients that fulfilled the criteria for overuse of medication, nor those receiving any preventive therapy, as it was our aim that our cohort would reflect a real clinical setting as closely as possible.

After having decided to initiate OnabotA therapy, patients were trained during one month to complete a diary where they recorded information on their headache days, migraine days (defined as high intensity, lateralized pain with a strong repercussion on daily activities) and the number of days on which they took acute headache medication, in particular triptans, as well as the number of visits to the emergency department as a consequence of headache. Three months after each treatment session, we considered how each variable collected in the diary had been modified. In all cases, OnabotA was administered according to the PREEMPT (Blumenfeld et al. 2010) protocol, performing no additional injections in the first two treatment sessions. We also asked patients to express their subjective consideration of the efficacy after each session (excellent, good, partial or no effect).

Statistical analysis was performed with the SPSS statistical package (version 20.0 Inc., Chicago, IL, USA), and any possible association between the baseline migraine data and those recorded after each treatment was assessed using a Student’s *t*-test.

## Results

By September 2014 we had treated 52 CM patients (8 male, 44 female) with OnabotA according to the PREEMPT protocol. The mean age of this cohort at migraine onset was 16.8 ± 7.8 years (range 3–32) and the mean age at treatment onset was 42.8 ± 12.7 years (16–71). At inclusion, 43 cases (82.7%) fulfilled the revised ICHD-II criteria for symptomatic overuse of medication (Olessen et al. 2006) and 44 (84.6%) were receiving preventive therapy. The migraine characteristics of these 52 patients are shown in Table 1.

**Table 1:** **Migraine characteristics at inclusion in the 52 patients**

**Variable**	
Overuse of symptomatic medication	43/52 (82.7%)
- Analgesics	31/52 (59.6%)
- Combined medications	12/52 (23.1%)
Previous use of neuromodulators	52/52 (100%)
- Topiramate	48/52 (92.3%)
- Other	26/52 (50%)
Previous use of beta-blockers	46/52 (88.5%)
Previous use of Calcium channel antagonists	33/52 (63.5%)
Previous use of antidepressants	30/52 (57.7%)
Previous use of anaesthetic blockades	11/52 (21.1%)
Monthly headache days	23.4 ± 6.3 (15–30)*
Monthly migraine days	13.9 ± 7.3 (8–30)*
Monthly symptomatic medication intake days	17.7 ± 9.2 (3–30)*
Monthly triptan intake days	5.1 ± 6.9 (0–25)*
Monthly visits to emergency department	0.25 ± 0.9 (0–6)*

*mean ± standard deviation (range).

The response to the first session of treatment in these 52 cases is shown in Table 2. Following treatment, there was a reduction of between 46.5 and 58.1% in the number of headache days, migraine days and days of acute medication or triptan intake. Indeed, the proportion of patients that experienced a reduction of at least 50% in any of these variables ranged between 57.6 and 74%. The parameter that diminished in the largest number of patients was the number of days of triptan intake. In all cases the differences were statistically significant (p ≤ 0.05).

**Table 2:** **Changes in the variables evaluated after first treatment cycle (n = 52 patients)**

**Variable**	**Before treatment**	**After treatment**	***P***	**Reduction**	**Reduction** ≥ **50%**
Headache days	23.4 ± 6.3*	12.8 ± 9.6*	<0.001	46.5%	30/52 (57.6%)
Migraine days	13.9 ± 7.3*	5.3 ± 5.5*	<0.001	52.9%	34/52 (65.3%)
Medication intake days	17.7 ± 9.2*	8.7 ± 8*	<0.001	50.4%	31/52 (59.6%)
Triptan intake days	5.1 ± 6.9*	2.1 ± 3.6*	<0.001	58.1%	20/27 (74%)

*mean ± standard deviation (range).

There were 12 patients (23.1%) who perceived a lack of efficacy after the first treatment cycle and although we encouraged them to accept a second treatment, four of them refused.

In total, 168 treatment cycles were administered and performing a second set of injections on 39 patients produced a significant reduction in all the variables assessed compared to the baseline (Table 3). The reduction in the number of days with pain or migraine, and in acute medication or triptan intake ranged between 36.3 and 73.1%. Moreover, the proportion of patients in whom the reduction was at least 50% in any of these parameters oscillated between 57.9 and 74.3%. These differences were statistically significant for all the variables measured, although the greatest reduction after the second cycle of OnabotA injections was achieved in the number of migraine days.

**Table 3:** **Changes in the variables evaluated between the baseline and three months after the second treatment cycle (n = 39 patients)**

**Variable**	**Before treatment**	**After treatment**	**P**	**Reduction**	**Reduction** ≥ **50%**
Headache days	23.8 ± 6.9*	9.2 ± 9.8*	*<0.001*	62%	27/39 (69.2%)
Migraine days	14.7 ± 7.6*	3.9 ± 6.4*	*<0.001*	73.1%	29/39 (74.3%)
Medication intake days	18.9 ± 9*	7.4 ± 8.2*	*<0.001*	57.7%	28/39 (71.8%)
Triptan intake days	5.4 ± 7.3*	2.3 ± 3.7*	*<0.001*	36.3%	11/19 (57.9%)

*mean ± standard deviation (range).

In no case was the treatment discontinued due to adverse effects.

## Discussion

Chronic migraine is diagnosed in 5% of patients that are referred to a general neurology department (Oterino et al. 2011) and globally, migraine is currently considered the seventh most influential disabling condition according to World Health Organization (Steiner et al. 2013). It is a condition that has an important negative impact on the life of an individual, particularly in terms of family and social interactions, as well as at the economic or occupational levels. Indeed, CM is associated with a substantial reduction in quality of life.

Though CM requires preventive therapy, only 33.3% of the patients referred to a headache unit had previously received such treatment (Mathew & Jaffri 2009). Topiramate is the only drug among the medications used in episodic migraine prophylaxis for which benefits in the treatment of CM have been established (Cady et al. 2011; Palma et al. 2012). However, up to 5% of CM patients attending a Headache unit are refractory to oral therapies (Aurora et al. 2011), and this group of patients includes those that are more disabled and that have a worse quality of life.

The PREEMPT (Phase III REsearch Evaluating Migraine Prophylaxis Therapy) clinical study was a multicenter, double-blind and placebo-controlled trial carried out in two phases. This study demonstrated the efficacy, safety and tolerability of OnabotA as a prophylactic treatment for CM in adults (Aurora et al. 2010; Diener et al. 2010; Dodick et al. 2010; Blumenfeld et al. 2010). However, the mechanisms by which OnabotA decreases the frequency and intensity of pain attacks in CM patients are not well understood.

In our series of patients, CM refractory to oral preventative therapies is more frequent among females, with an onset of episodic migraine around the second decade of life and a need for OnabotA therapy in the beginning of fifth decade. These data are similar to those described previously and they show that there may be a long latency period between the onset of migraine and the need for OnabotA therapy. This could indicate that CM is in some cases part of the natural evolution of episodic migraine (Álvaro-González et al. 2012), for which there is currently no conclusive evidence regarding the efficacy of treatment with OnabotA (Aurora et al. 2007; Relja et al. 2007).

The baseline situation of the patients in our series was similar to that described in the PREEMPT (Dodick et al. 2010) trial, as well as that in recently published observational studies (Palma et al. 2012; Álvaro-González et al. 2012; Silberstein et al. 2013). We considered oral preventative refractoriness as an inclusion criteria, including the failure of topiramate therapy (or other neuromodulators if topiramate is not tolerated) and at least one other preventative therapy (beta blockers and/or calcium channel antagonists). Indeed, new consensus criteria has included refractoriness to OnabotulinumtoxinA among the chronic migraine criteria (Martelletti et al. 2014).

In this study we present data regarding the response to a first cycle of OnabotA therapy, a situation rarely considered in the literature, especially when employing the PREEMPT paradigm (Álvaro-González et al. 2012). Our results are more homogeneous as we did not administer the remaining 40 IU to the additional areas where pain was experienced as considered in the first two treatment sessions of the PREEMPT protocol. Rather, we reserved these additional units for patients whose response time was shorter than three months after second session.

The response rate was considered as the reduction of headache days, as in the PREEMPT clinical program (at least half of them (Dodick et al. 2010)), and it is above 50%. As described in other series, greater reductions are achieved in the number of migraine days and the number of days of triptans intake (Aurora et al. 2010; Oterino et al. 2011; Lipton et al. 2011). In our series, the subjective evaluation of efficacy referred to by the patients was between excellent and partial in most of them. These responses imply an important improvement in quality of life, as noted previously in the literature (Khalil et al. 2014; Batty et al. 2013).

The safety and tolerability of OnabotA was excellent in our population, with only mild pain at the injection site reported in some cases, and a mild dysphagia in one female patient with a low body mass index, which did not recur after the 4 paracervical injections were excluded in the following cycle. Therefore, as described in both the PREEMPT trial and in open studies, adverse effects of OnabotA in CM are scarce and reversible (Aurora et al. 2010; Blumenfeld et al. 2010; Álvaro-González et al. 2012; Aurora et al. 2007).

Health service approval of OnabotA as a prophylactic treatment for CM has allowed us to offer this therapy to our patients, although as with any new indication, clinicians must remain vigilant about its safety and efficacy (Jackson et al. 2012). Further studies are needed to answer some questions that remain open, such as the existence of response predictors (including biomarkers), the management of concomitant oral preventative therapies, or the possible need to modify the OnabotA paradigm depending on the type of response, only partial or good and sustained.

## Conclusion

In conclusion, when used according to the PREEMPT paradigm OnabotA is a safe and effective treatment in a real clinical setting, even when criteria of refractoriness to oral preventatives exist.
